# Supplementary figures and images for: Changes in Body Composition During Intensive Care Unit Stay and Outcomes in Patients with Severe COVID-19 Pneumonia: A Retrospective Cohort Study
Source: Viruses. 2025 Apr 29;17(5):643. doi: 10.3390/v17050643 (PMC12115843; doi:10.3390/v17050643)

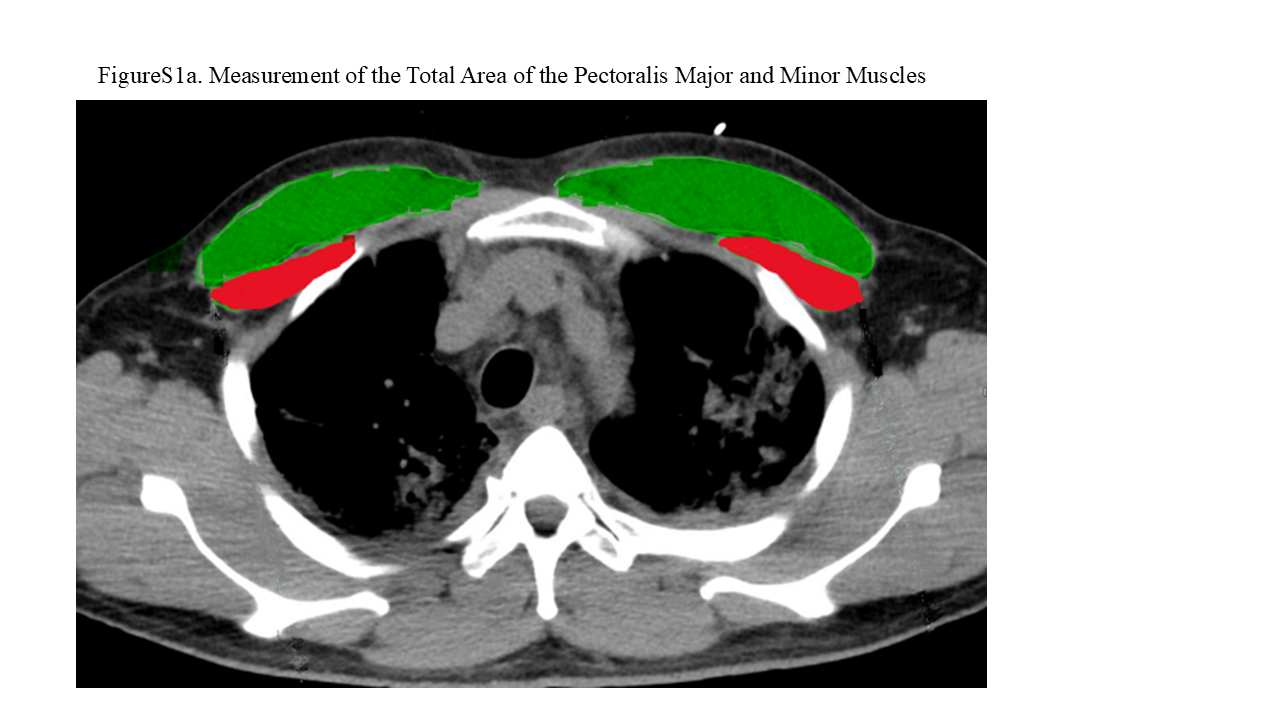

Supplement: Supplementary file 1 [file viruses-17-00643-s001.zip › FigureS1a.TIF]

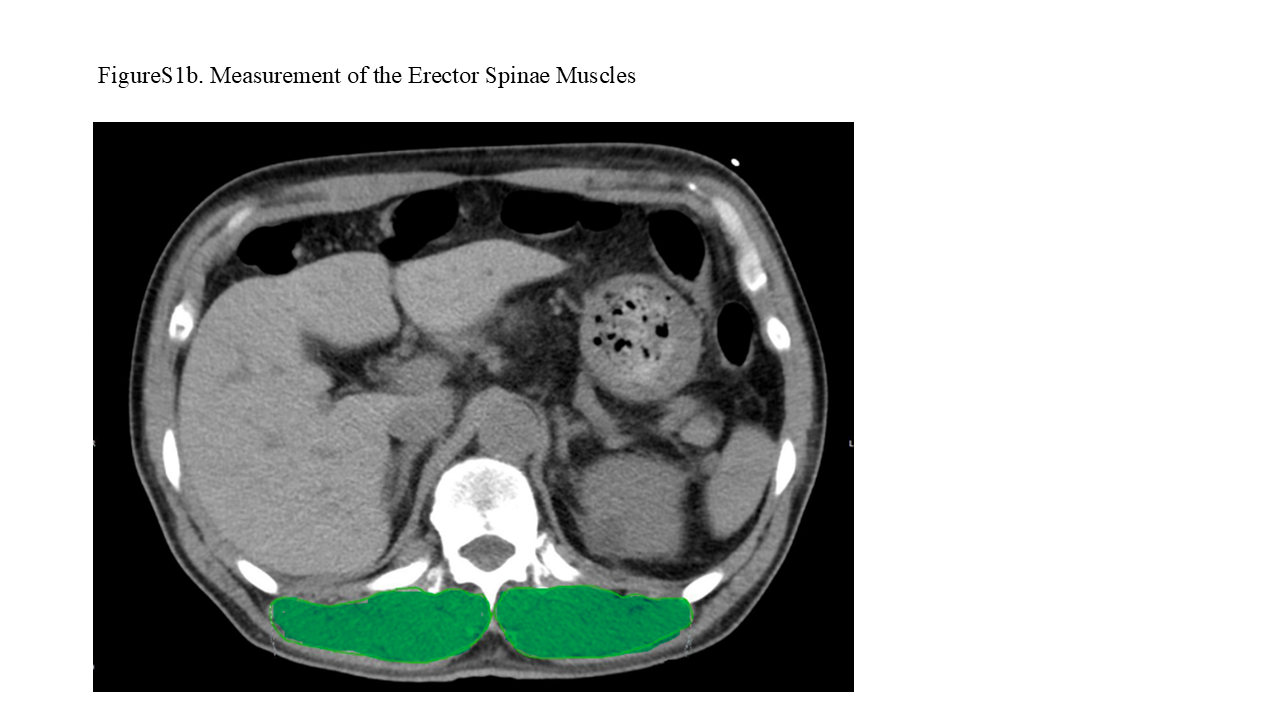

Supplement: Supplementary file 1 [file viruses-17-00643-s001.zip › FigureS1b.TIF]

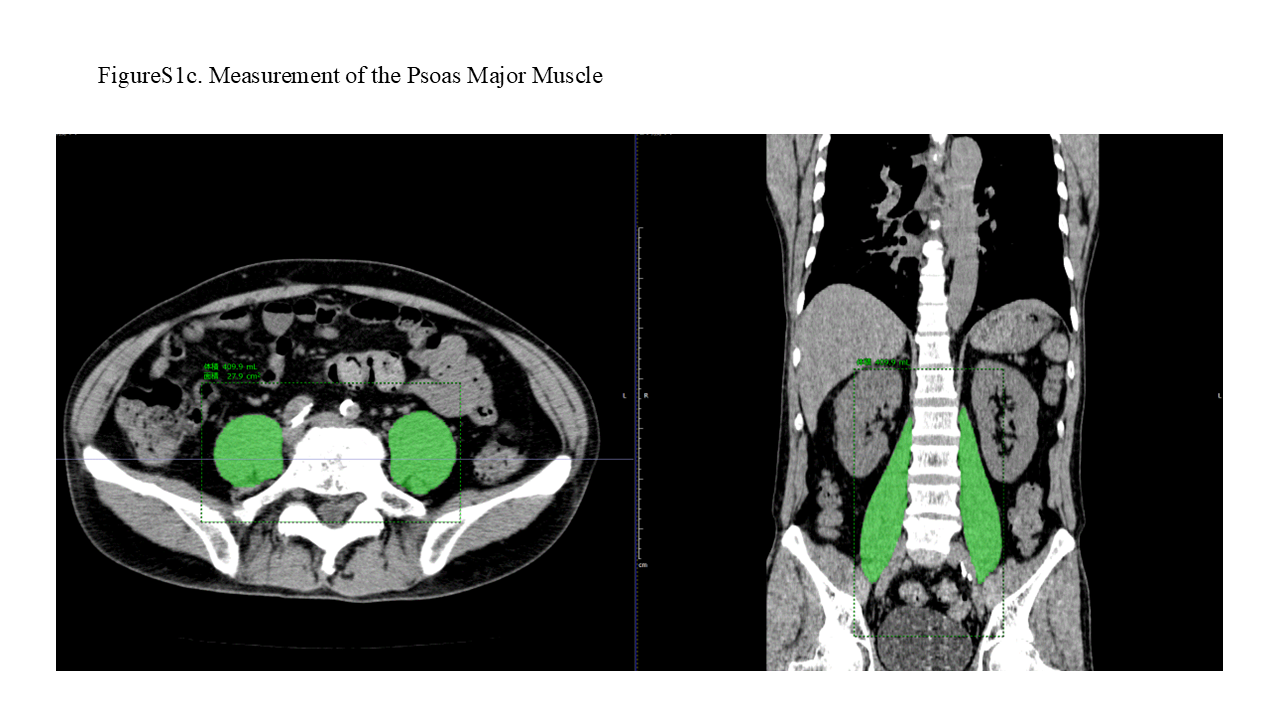

Supplement: Supplementary file 1 [file viruses-17-00643-s001.zip › FigureS1c.TIF]

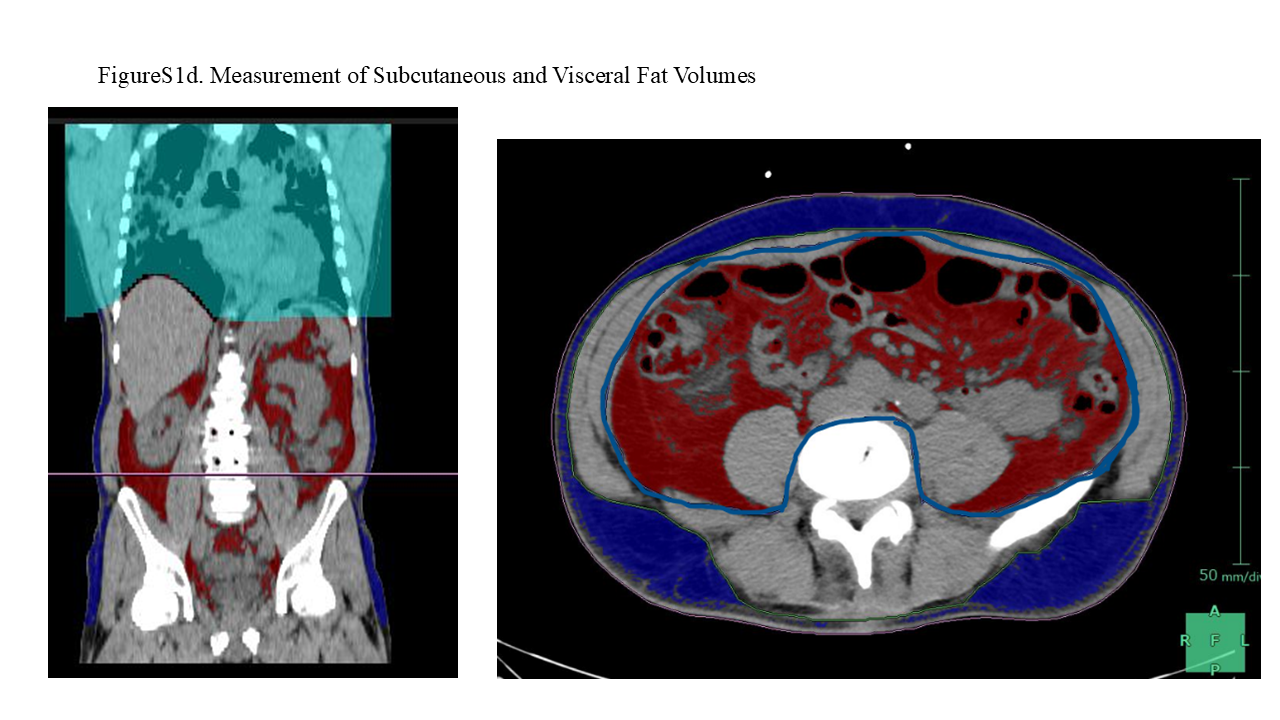

Supplement: Supplementary file 1 [file viruses-17-00643-s001.zip › FigureS1d.TIF]

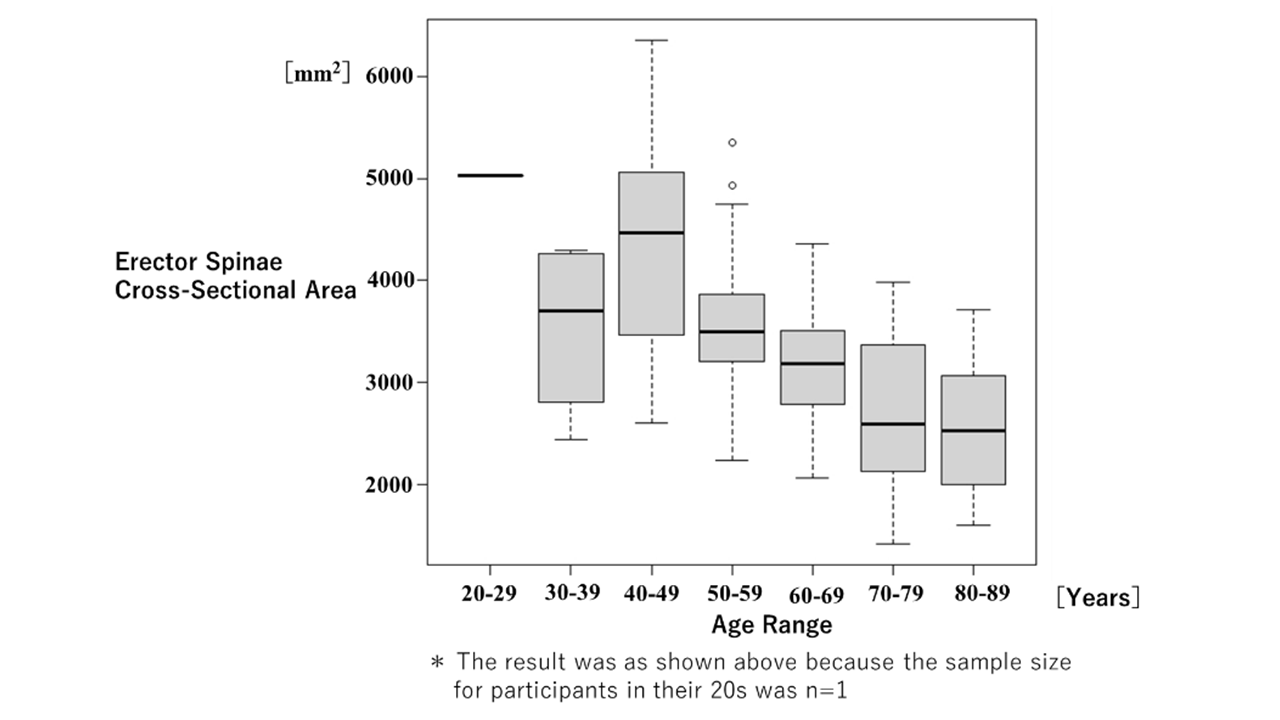

Supplement: Supplementary file 1 [file viruses-17-00643-s001.zip › FigureS2a.TIF]

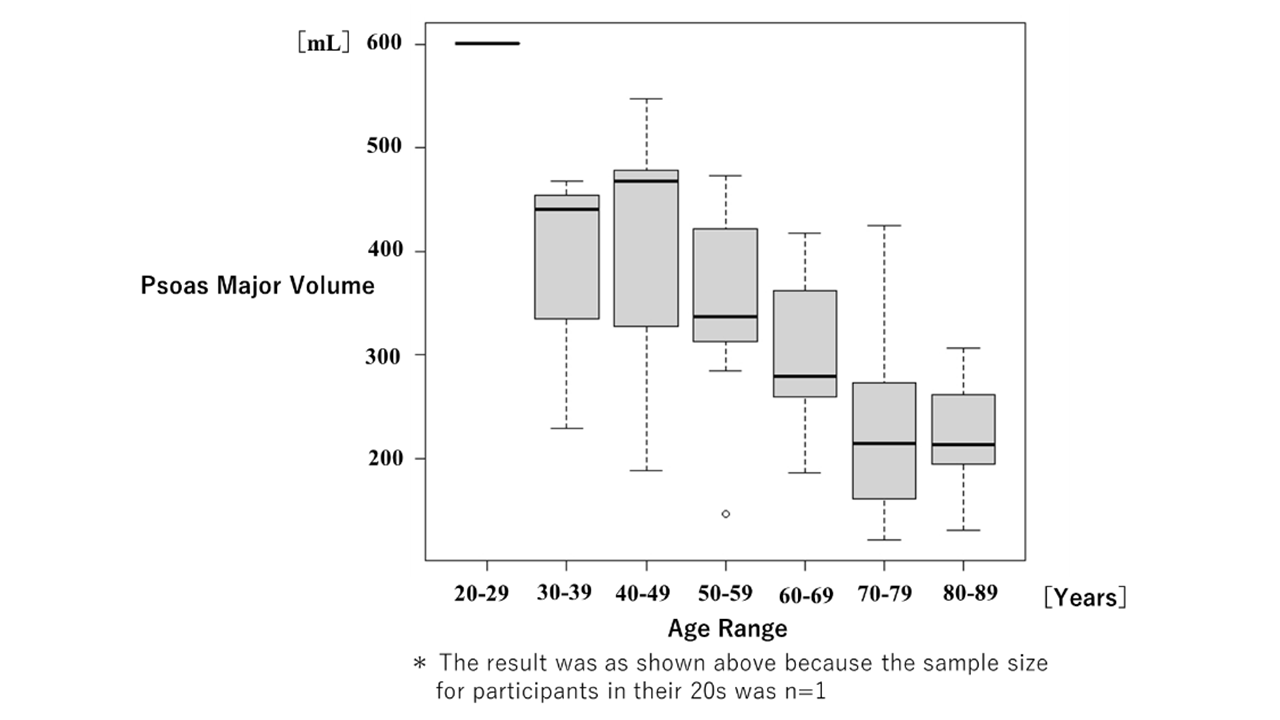

Supplement: Supplementary file 1 [file viruses-17-00643-s001.zip › FigureS2b.TIF]

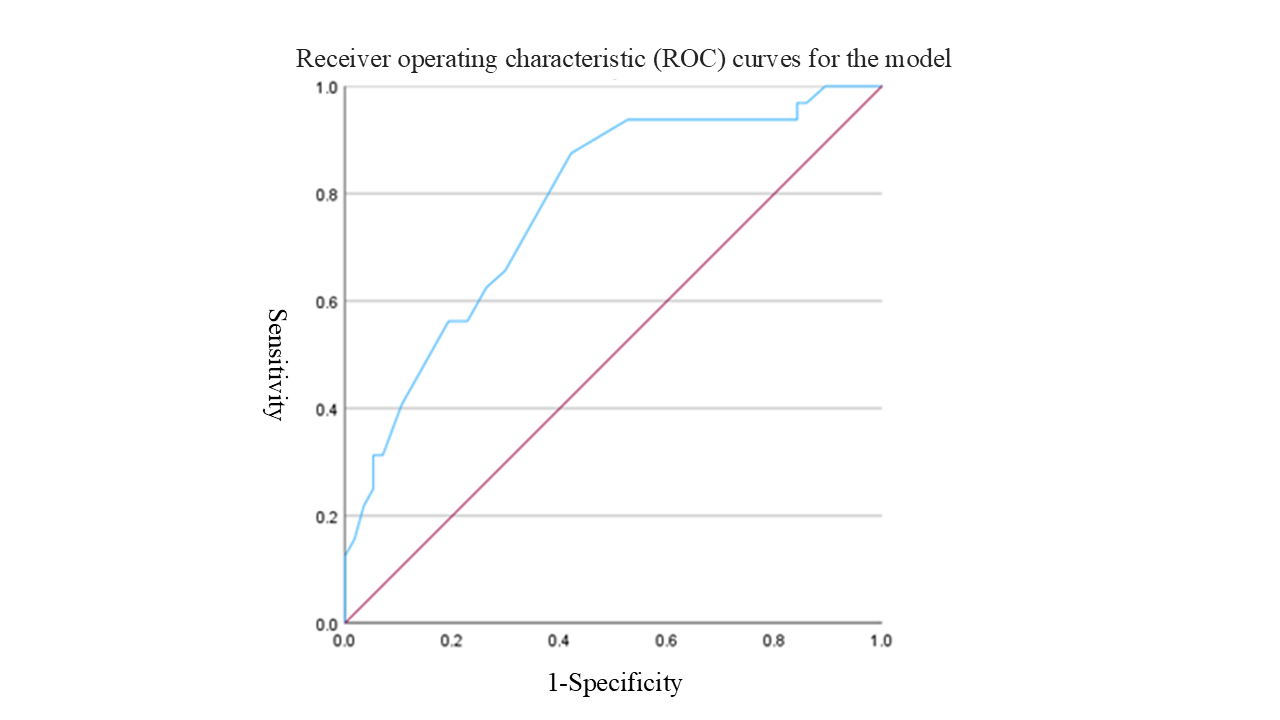

Supplement: Supplementary file 1 [file viruses-17-00643-s001.zip › Figure S3.tif]
